# The Anti-Inflammatory Potential of Levosimendan in Sepsis: An Experimental Study Using a LPS-Induced Rat Model

**DOI:** 10.3390/life15060928

**Published:** 2025-06-09

**Authors:** Elif Dedeler Ertanıdır, Ipek Duman, Duygu Onmaz Eryavuz, Ali Ünlü, Mehmet Ertanıdır, Ateş Duman

**Affiliations:** 1 Department of Anesthesiology and Reanimation, Faculty of Medicine, Selcuk University, Konya 42130, Türkiye; drelifdedeler@gmail.com (E.D.E.); ates.duman@atlas.edu.tr (A.D.); 2 Department of Medical Pharmacology, Faculty of Medicine, Necmettin Erbakan University, Konya 42080, Türkiye; 3 Department of Biochemistry, Faculty of Medicine, Bandirma Onyedi Eylül University, Bandırma, Balıkesir 10200, Türkiye; donmez@bandirma.edu.tr; 4 Department of Biochemistry, Faculty of Medicine, Selcuk University, Konya 42130, Türkiye; aunlu@selcuk.edu.tr; 5 Department of Orthopedics and Traumatology, Midyat State Hospital, Mardin 65420, Türkiye; m.ertanidir@hotmail.com

**Keywords:** cytokines, inflammation, levosimendan, sepsis, rat

## Abstract

Sepsis is a life-threatening condition driven by a dysregulated host immune response to infection, with cytokine overproduction contributing to organ dysfunction and high mortality. Levosimendan, a calcium sensitizer used in acute heart failure, has been proposed to exert anti-inflammatory effects, but information on its immunomodulatory effects in early sepsis remains scarce. This study aimed to investigate the dose- and time-dependent effects of levosimendan on cytokine profiles in a rat model of lipopolysaccharide (LPS)-induced sepsis. Thirty-two male Wistar albino rats were randomly assigned to four groups: sham, sepsis control, low-dose levosimendan (1 mg/kg), and high-dose levosimendan (2 mg/kg). Cytokine levels (TNF-α, IL-1β, IL-6, IL-8, IL-17, MCP-1) were measured at 5 and 10 h post-LPS administration. High-dose levosimendan significantly reduced TNF-α, IL-1β, IL-6, and MCP-1 levels by the 10th hour, accompanied by improved Murine Sepsis Scores. IL-17 and IL-6 showed biphasic responses, increasing initially and decreasing significantly later, particularly with high-dose treatment. IL-8 reduction was observed only in the high-dose group. These findings support levosimendan’s dose and time-dependent anti-inflammatory effects and suggest it may modulate both early and late-phase cytokines in sepsis. Further studies are warranted to clarify its potential role in clinical sepsis management.

## 1. Introduction

Sepsis is defined as life-threatening organ dysfunction caused by a dysregulated host response to infection [1]. Sepsis and resulting septic shock are the leading causes of intensive care unit admission and mortality among critically ill patients. Up to 25% of annual hospitalizations are due to sepsis, with mortality rates ranging from 15 to 56%. If adequate treatment is not administered in time, mortality can reach 70 to 90% [2,3,4].

**Figure 1:**
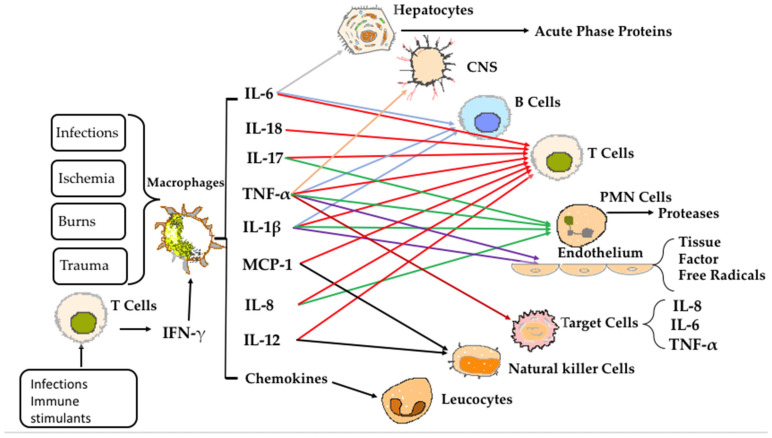
Schematic representation of the main pro-inflammatory cytokine regulation.

The complex pathophysiology of sepsis remains a topic of active debate and investigation. Cytokines play a central role in both the pathogenesis of sepsis and the associated inflammatory processes. These are small proteins primarily secreted by immune cells that coordinate immune responses (Figure 1).

Recent studies have highlighted the role of cytokine networks in sepsis progression. Each cytokine exhibits a distinct release and activity profile at different stages of sepsis, typically peaking and declining at different time points following sepsis induction. During the early phases of sepsis, pro-inflammatory cytokines such as tumor necrosis factor-alpha (TNF-α), interleukin-1β (IL-1β), and interleukin-6 (IL-6) are released, initiating the inflammatory cascade [5]. TNF-α is one of the earliest cytokines to rise, generally peaking within 1 to 2 h after the onset of sepsis and returning to baseline levels within 4 to 6 h [5]. IL-1β mRNA levels peak approximately 3 to 4 h after endotoxin stimulation, remain elevated for 6 to 8 h, and then decline rapidly [6]. IL-6 typically peaks between 4 and 6 h post-induction and may remain elevated longer than TNF-α, sometimes persisting for 12 to 24 h or even several days in severe cases [7]. Elevated levels of IL-8 are associated with poorer outcomes in septic patients, as excessive neutrophil activation exacerbates tissue and organ injury [8]. IL-8 levels increase rapidly within 1 to 3 h of inflammation onset. Given its short half-life of less than 24 h, IL-8 concentrations can decline swiftly following effective antibiotic treatment [9]. Interleukin-17 (IL-17) appears to have a dual role: while some studies emphasize its pro-inflammatory actions, others underscore its potential protective effects. IL-17A, generally peaks later—around 12 to 24 h after sepsis initiation. It plays a key role in amplifying inflammatory signaling, thereby contributing to tissue damage and prolonged immune activation in sustained sepsis [9,10,11]. Monocyte Chemoattractant Protein-1 (MCP-1), secreted by monocytes, endothelial cells, fibroblasts, and smooth muscle cells in response to inflammatory signals, plays a key role in attracting monocytes, macrophages, and T cells to sites of inflammation. MCP-1 facilitates prolonged immune cell recruitment and contributes to the cytokine storm observed in sepsis, promoting immune dysregulation and organ damage. MCP-1 levels often rise moderately during the early phase, peaking slightly later—around 6 to 12 h—and may remain elevated for an extended duration, particularly if inflammation persists, occasionally lasting up to 24 h or more [12,13]. Conversely, anti-inflammatory cytokines—such as interleukin-10 (IL-10)—act to restrain excessive inflammation. However, they may also lead to immune suppression, rendering patients more susceptible to secondary infections and adverse outcomes [14].

Murine sepsis models are widely utilized to investigate cytokine dynamics during sepsis, as well as the effects of various therapeutic agents on cytokine responses [15,16]. These models replicate the cytokine storm observed in human sepsis and facilitate the study of interventions that specifically target cytokine-mediated pathways [17]. Administration of exogenous toxins such as lipopolysaccharide (LPS), the fecal sepsis model, or models involving the infusion or inoculation of exogenous bacteria are the most commonly employed models [18]. LPS is a glycolipid component of the outer membrane of Gram-negative bacteria and induces systemic inflammation by triggering the release of inflammatory cytokines, thereby mimicking many clinical features of sepsis. It is administered either intraperitoneally or intravenously. In the fecal sepsis model, a fecal emulsion is directly injected into the abdominal cavity, leading to widespread peritoneal contamination. In models involving the infusion or inoculation of exogenous bacteria, live bacteria are injected either into the peritoneal cavity or directly into the bloodstream. The LPS model of sepsis remains highly relevant in experimental sepsis research, especially for evaluating inflammation-targeted therapies and cytokine modulation strategies. This immune cascade closely mirrors the cytokine storm and organ damage characteristic of clinical sepsis [15,19,20].

Levosimendan is a calcium-sensitizing inotropic agent that enhances myocardial contractility and is primarily used in the management of acute decompensated heart failure. Emerging evidence suggests that levosimendan also exhibits anti-apoptotic, antioxidant, and anti-inflammatory properties, which may contribute to the preservation of renal and cardiac function in patients with heart failure [21,22]. In the context of septic shock, both inotropic support and the preservation of myocardial cell integrity are critical. Due to its anti-inflammatory effects, levosimendan may offer an additional therapeutic benefit in the treatment of sepsis and septic shock. Levosimendan has been shown to modulate systemic inflammatory responses, potentially mitigating the progression of organ failure and reducing mortality [22].

This study was designed to investigate the effects of levosimendan on the early progression of sepsis and cytokine production, specifically within the first 10 h, using a LPS-induced sepsis model in rats.

## 2. Materials and Methods

### 2.1. Animal Model

All animal procedures adhered to the European Community guidelines (2010/63/EU) [23] and the ARRIVE guidelines for animal experiments [24]. The Institutional Animal Experiments Ethics Committee approved the study. Thirty-two male Wistar albino rats (3–6 months old, 450–550 g) were housed under standard laboratory conditions: 12-h light/dark cycle, room temperature, with free access to dry food pellets and water.

Using block randomization, rats were randomly assigned to four groups (*n* = 8 per group). Sham group (A): No interventions; baseline cytokine levels were assessed in healthy animals. Sepsis control group (B): Received an intraperitoneal (i.p.) injection of LPS at 5 mg/kg to induce sepsis; no subsequent treatment. Low-dose levosimendan group (C): Sepsis induced with LPS (5 mg/kg i.p.), followed by levosimendan administration (1 mg/kg i.p.) 2 h later. High-dose levosimendan group (D): Sepsis induced with LPS (5 mg/kg i.p.), followed by levosimendan administration (2 mg/kg i.p.) 2 h later.

Five and ten hours post-LPS administration, groups B, C, and D were assessed using the Murine Sepsis Score (MSS). Group A was evaluated prior to blood sampling. The MSS evaluates multiple physiological and behavioral parameters such as general appearance, activity level, respiratory distress, eye and skin changes, and body temperature. Each parameter is scored numerically from 0 to 4, with higher scores indicating more severe sepsis progression [25].

### 2.2. Sampling Method

Blood collection and euthanasia were conducted under general anesthesia. Anesthesia was induced with xylazine hydrochloride (10 mg/kg) and ketamine (50 mg/kg) administered intraperitoneally.

Sham group (A): Intracardiac blood samples were collected once, followed by euthanasia via cervical dislocation. Sepsis groups (B, C, D): Tail vein blood samples (1 mL) were collected 5 h post-LPS administration. Intracardiac blood samples were obtained 10 h post-LPS, followed by euthanasia. Samples were centrifuged at 3000 rpm for 10 min, and sera were stored at −80 °C until analysis. The maximum storage time for sera was 10 days.

### 2.3. Cytokine Assay

Serum levels of TNF-α, IL-1β, IL-6, IL-8, IL-17, and MCP-1 were quantified using enzyme-linked immunosorbent assay (ELISA) kits, following the manufacturer’s protocols. Chemicals and Reagents: LPS (055:B5): Sigma-Aldrich, St. Louis, MO, USA, Levosimendan (3001077): Abbott Laboratories, Green Oaks, IL, USA, ELISA Kits: BT LAB, Shanghai, China.

### 2.4. Statistical Analysis

Analyses were performed using the R 4.4.2 (R Core Team, 2024) program. Data are presented as the mean ± standard deviation. For all outcomes of the study, mixed effects models with random intercepts including group and time-fixed effects with their interaction were used. Least squares means comparisons with Tukey adjustments post-hoc were performed. *p* < 0.05 was considered significant.

## 3. Results

### 3.1. Murine Sepsis Score (MSS)

**Figure 2:**
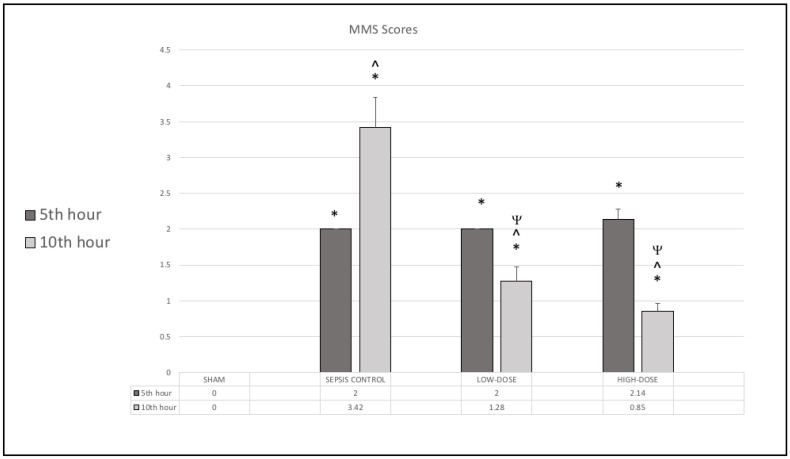
Murine Sepsis Scores (MSS) of sham, sepsis control, low-dose levosimendan, and high-dose levosimendan groups. * *p* < 0.05 compared to the sham group, ^ *p* < 0.05 compared to 5th hour. ^Ψ^ *p* < 0.05 compared to the sepsis control group.

The rats were assessed for sepsis-related clinical manifestations using the Murine Sepsis Score. At the 5th hour, MSS values were significantly elevated in all LPS-treated groups compared to the sham group (*p* < 0.05), indicating the presence of systemic inflammation. By the 10th hour, the MSS score had further increased in the sepsis control group. However, both levosimendan-treated groups showed a significant reduction in MSS scores at the 10th hour, with the high-dose group demonstrating the most pronounced improvement in MSS scores (Figure 2).

### 3.2. Cytokine Levels

Cytokine concentrations were measured at the 5th and 10th hours following LPS administration to evaluate the immunomodulatory effects of levosimendan. The temporal profiles and intergroup differences for each cytokine are presented below.

#### 3.2.1. TNF-α

**Figure 3:**
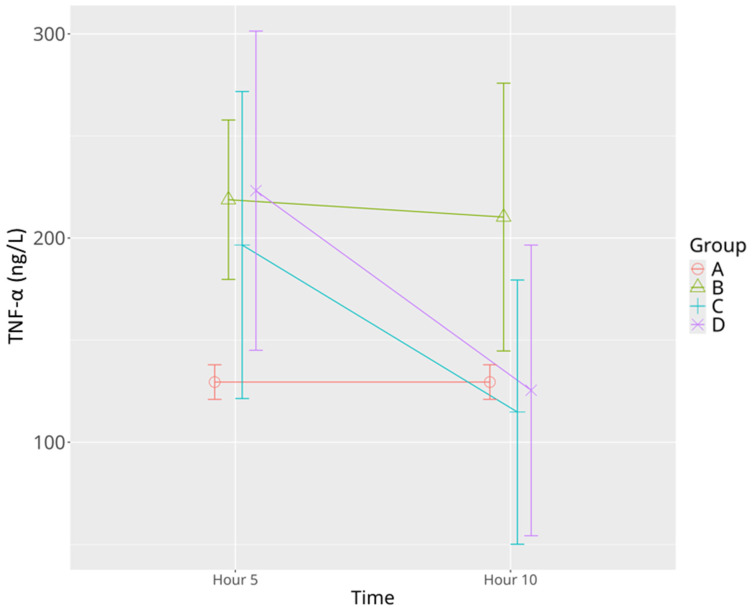
Effects of levosimendan on TNF-α levels. Values are mean ± SD (ng/L). Each group (*n* = 8). Red: sham (group A); green: sepsis (group B); blue: low-dose levosimendan (group C); purple: high-dose levosimendan (group D).

A significant increase in TNF-α was observed in the sepsis control group at both the 5th and 10th hours compared to the sham group, indicating a sustained inflammatory response to LPS. Low-dose levosimendan (Group C) did not significantly alter TNF-α levels at the 5th hour relative to either the sham or sepsis control groups; however, a significant reduction was observed at the 10th hour, both in comparison to its own 5th-hour values and to the 10th-hour levels of the sepsis control group. The high-dose levosimendan (Group D) was associated with a marked elevation in TNF-α at the 5th hour, significantly higher than the sham group, followed by a substantial decline at the 10th hour. At this later time point, TNF-α levels in the high-dose group were comparable to those of the sham group, reflecting a time- and dose-dependent anti-inflammatory effect (Table 1, Figure 3). Mixed model analysis demonstrated significant effects of time (*p* = 0.003), group (*p* = 0.035), and a trend toward significance for time–group interaction (*p* = 0.051).

#### 3.2.2. IL-1β 

**Figure 4:**
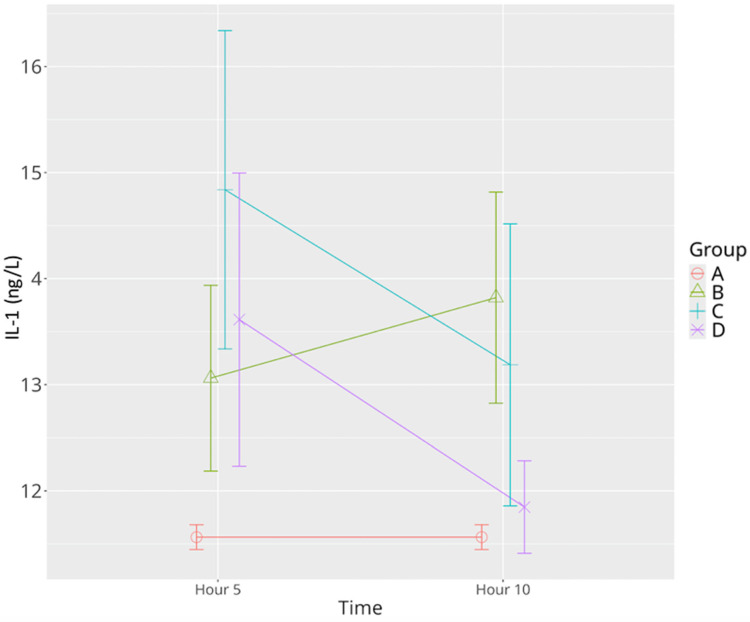
Effects of levosimendan on IL-1β levels. Values are mean ± SD (ng/L). Each group (*n* = 8). Red: sham (group A); green: sepsis (group B); blue: low-dose levosimendan (group C); purple: high-dose levosimendan (group D).

IL-1β concentrations in the sepsis control group increased progressively, with a statistically significant elevation at the 10th hour relative to the sham group. In the low-dose levosimendan group, IL-1β levels were significantly elevated at the 5th hour, exceeding those observed in both the sham and sepsis control groups. Although a significant decrease was observed by the 10th hour, values remained higher than those of the sham group. High-dose levosimendan also induced a significant elevation at the 5th hour; however, by the 10th hour, IL-1β levels had returned to levels not significantly different from the sham group, suggesting a more potent anti-inflammatory action at higher doses (Table 1, Figure 4). Mixed model analysis confirmed significant main effects for time (*p* = 0.032), group (*p* < 0.001), and time–group interaction (*p* = 0.011).

#### 3.2.3. IL-6

**Figure 5:**
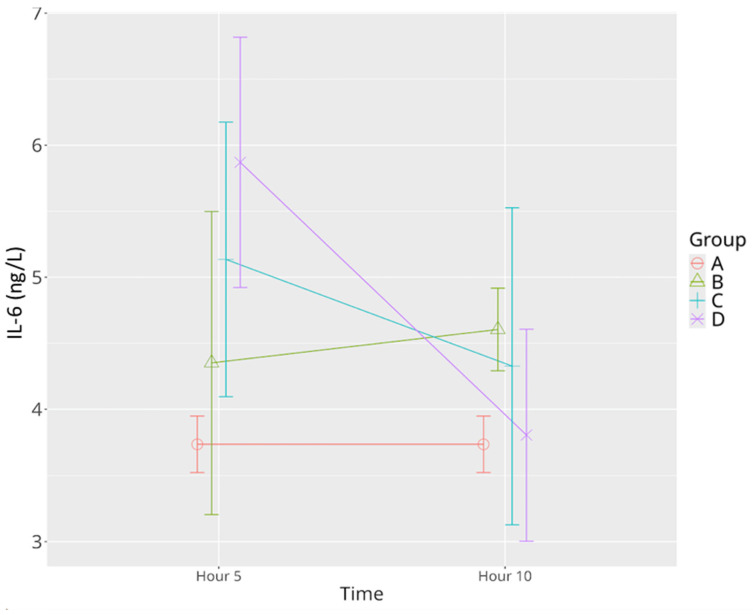
Effects of levosimendan on IL-6 levels. Values are mean ± SD (ng/L). Each group (*n* = 8). Red: sham (group A); green: sepsis (group B); blue: low-dose levosimendan (group C); purple: high-dose levosimendan (group D).

The sepsis control group demonstrated a significant increase in IL-6 concentrations over time, with higher levels observed at the 10th hour. Administration of low-dose levosimendan resulted in elevated IL-6 levels at the 5th hour, followed by a non-significant decrease by the 10th hour. Notably, this late-phase reduction brought levels statistically in line with the sham group. High-dose levosimendan elicited a pronounced rise in IL-6 at the 5th hour, significantly higher than both the sham and sepsis control groups. By the 10th hour; however, IL-6 concentrations had significantly decreased, aligning closely with those observed in the sham group (Table 1, Figure 5). The analysis revealed significant effects of time (*p* = 0.008), group (*p* = 0.031), and time–group interaction (*p* = 0.005).

#### 3.2.4. IL-8

**Figure 6:**
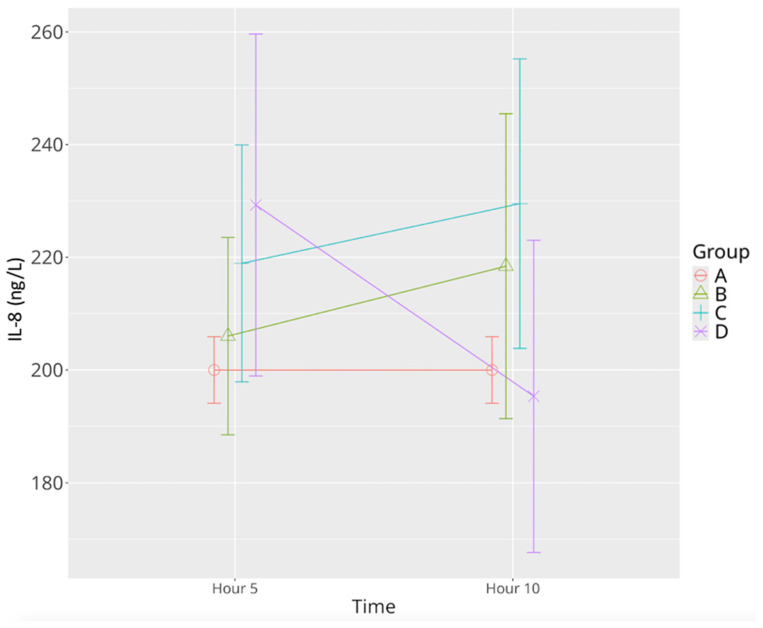
Effects of levosimendan on IL-8 levels. Values are mean ± SD (ng/L). Each group (*n* = 8). Red: sham (group A); green: sepsis (group B); blue: low-dose levosimendan (group C); purple: high-dose levosimendan (group D).

IL-8 levels in the sepsis control group showed a non-significant upward trend between the 5th and 10th hours. Similar patterns were noted in both levosimendan-treated groups. However, only the high-dose group exhibited a significant reduction between the 5th and 10th hours (Table 1, Figure 6). Although overall differences among groups were not significant, a significant time–group interaction was detected (*p* = 0.025), indicating a differential cytokine response trajectory influenced by the treatment modality.

#### 3.2.5. IL-17

**Figure 7:**
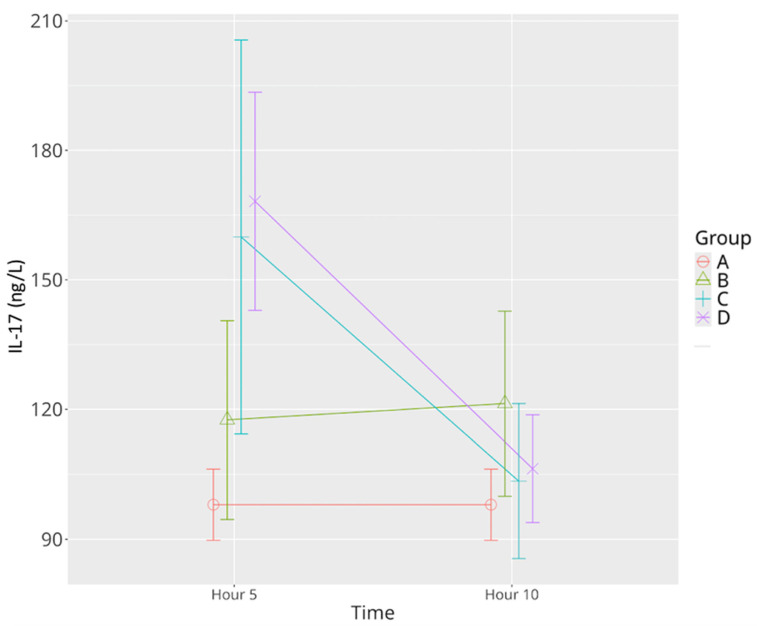
Effects of levosimendan on IL-17 levels. Values are mean ± SD (ng/L). Each group (*n* = 8). Red: sham (group A); green: sepsis (group B); blue: low-dose levosimendan (group C); purple: high-dose levosimendan (group D).

In the sepsis control group, IL-17 concentrations increased slightly but not significantly over time. Both low- and high-dose levosimendan treatment resulted in a significant increase in IL-17 at the 5th hour compared to the sham and sepsis groups. By the 10th hour, a marked decline in IL-17 was observed in both treatment groups, with levels returning to values not significantly different from the sham group. This biphasic pattern suggests that levosimendan exerts a transient immunoactivating effect followed by a robust resolution of inflammation (Table 1, Figure 7). Statistical analysis confirmed significant effects for time (*p* < 0.001), group (*p* = 0.006), and time–group interaction (*p* < 0.001).

#### 3.2.6. MCP-1

**Figure 8:**
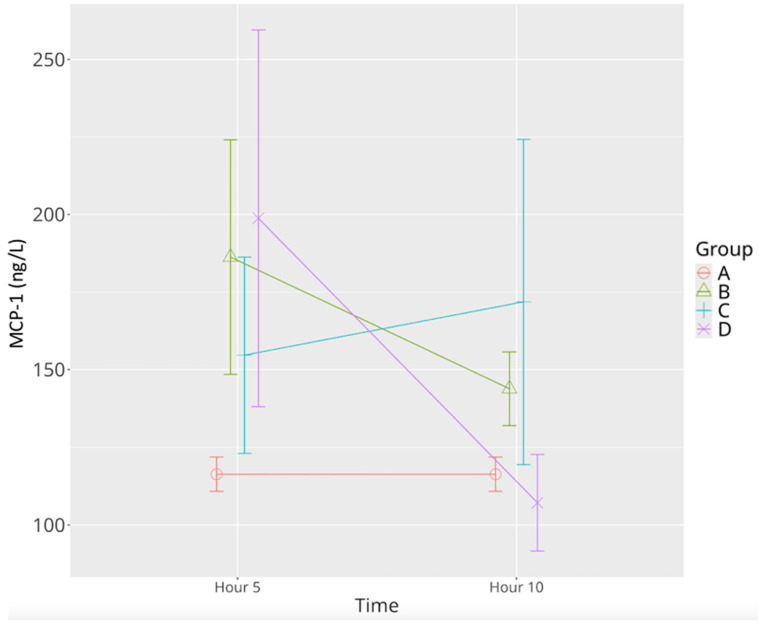
Effects of levosimendan on MCP-1 levels. Values are mean ± SD (ng/L). Each group (*n* = 8). Red: sham (group A); green: sepsis (group B); blue: low-dose levosimendan (group C); purple: high-dose levosimendan (group D).

MCP-1 levels were significantly elevated in the sepsis control group at the 5th hour compared to the sham group and remained moderately elevated at the 10th hour. In the low-dose levosimendan group, MCP-1 concentrations were significantly increased at both time points relative to the sham group, showing limited regulatory effect. In contrast, high-dose levosimendan induced a substantial elevation at the 5th hour followed by a sharp decline at the 10th hour, bringing levels below those of the sham group. These findings underscore a strong dose-dependent and temporally dynamic regulation of MCP-1 Table 1, Figure 8). The mixed model revealed significant effects for time (*p* = 0.006), group (*p* = 0.004), and time–group interaction (*p* = 0.002).

## 4. Discussion

This study reinforces and expands upon existing evidence of levosimendan’s anti-inflammatory effects by demonstrating its dose-dependent regulation of cytokine levels in early-stage experimental sepsis. Notably, high-dose levosimendan markedly reduces TNF-α, IL-1β, IL-6, and MCP-1 concentrations by the 10th hour and alleviates clinical severity, as reflected in decreased MSS scores. Furthermore, by incorporating IL-17 dynamics, the study provides additional insight into the broader cytokine modulation profile of levosimendan.

Our findings are consistent with those of Wang et al. [26], who observed that levosimendan reduced cytokine levels in septic models. They investigated the ability of levosimendan to suppress LPS-induced cytokine overproduction in primary peritoneal macrophages isolated from mice. LPS stimulation led to a significant increase in TNF, IL-6, and MCP-1 mRNA expression, which was markedly reduced following levosimendan treatment. ELISA analysis further confirmed a decrease in the secretion of these pro-inflammatory and chemotactic cytokines from LPS-stimulated macrophages. In addition to TNF-α, IL-1β, IL-6, and MCP-1, our in vivo work provides further insights by incorporating IL-17 and IL-8 responses. Our study also shows that IL-6 and IL-17 may transiently increase post-treatment before decreasing, a biphasic response not highlighted in earlier models. While most studies highlight the anti-inflammatory effects of levosimendan, explicit evidence of a dose-dependent relationship is limited. Our findings demonstrate a dose-dependent reduction in pro-inflammatory cytokines with levosimendan treatment, particularly at higher doses, supporting the hypothesis of a dose-dependent anti-inflammatory effect. Despite an early-phase increase in certain cytokines, higher doses of levosimendan were more effective in reducing IL-1β, MCP-1, and IL-6 cytokine levels compared to lower doses. Notably, levosimendan accelerated the resolution of IL-6, IL-1β, and IL-17, with high-dose treatment leading to substantial suppression by the 10th hour for IL-1β and IL-6. MCP-1, another late-phase cytokine involved in chemotaxis, also followed this trend, with its levels dropping below baseline in the high-dose group. IL-6 and IL-17 levels increased during the early phase at both doses of levosimendan; however, in the later phase, these cytokines returned to levels comparable to those of the sham group. This biphasic response suggests that levosimendan initially triggers immune activation, followed by a strong resolution of inflammation. IL-8 levels showed a non-significant upward trend in both the sepsis control and levosimendan-treated groups. However, a significant reduction was observed only in the high-dose levosimendan group between the 5th and 10th hours. On the other hand, in our study, our results showed that TNF-α decreased significantly with low-dose levosimendan as well as the high dose.

In an acute liver injury model, Sakaguchi et al. [19] found that pretreatment with both 2 and 4 mg/kg, i.p. of levosimendan improved the survival of rats. The higher-dose levosimendan had the most pronounced effects on lowering TNF-α, IL-6, and IL-1β levels, confirming our dose-dependent observations. The study showed a dose-dependent response in which higher doses of levosimendan had more pronounced effects on lowering early cytokine levels, especially TNF-α. The anti-inflammatory action was most evident within the first few hours, which they hypothesized could be due to levosimendan’s rapid vasodilatory and cellular protective effect. The differences with the present study may be due to the administration of levosimendan before the induction of sepsis. However, both results show higher dosing may be more effective in achieving optimal anti-inflammatory effects in early sepsis treatment. Beyond the sepsis model, Ateş et al. [27] demonstrated that higher doses of levosimendan effectively reduced IL-1 and IL-6 levels following blunt chest trauma in rats. Both low and high doses were administered, but the reduction in cytokine levels was significantly more pronounced with the high dose. These findings suggest that levosimendan’s anti-inflammatory effects are not limited to sepsis but also extend to trauma-induced inflammation, with higher doses showing greater efficacy in mitigating cytokine responses associated with tissue injury.

In our study, each cytokine exhibited a specific temporal response pattern. Statistical analysis confirmed significant effects for time and time–group interactions for most cytokines, which is consistent with the findings of Samuelsen et al. [28], who reported time-dependent variations in cytokine release from ex vivo-stimulated whole blood, including changes in monocyte Human Leukocyte Antigen-DR (mHLA-DR) expression in septic patients. Krychtiuk et al. [29] demonstrated that levosimendan maintains suppression of IL-6 and IL-8 levels beyond the acute phase, indicating potential long-term anti-inflammatory effects. In vitro, levosimendan significantly attenuated IL-1β-induced mRNA upregulation of IL-6 and IL-8. Comparable reductions were observed when cells were pre-treated with TNF-α, as levosimendan significantly decreased IL-6 and IL-8 expression. Additionally, IL-1β triggered a time-dependent increase in IL-6 and IL-8 protein levels, as confirmed by ELISA. The study demonstrated a long-term anti-inflammatory effect of levosimendan, reducing IL-6 and IL-8 levels beyond the immediate administration phase. This suggests that levosimendan may have lasting benefits in suppressing inflammation. Our findings show that, compared to the sham group, non-significant increases in IL-8 levels were observed in both levosimendan groups at both time points, similar to the sepsis control group. However, a significant decrease was noted between the 5th and 10th hours in the high-dose group. It should be noted that the study duration may have been limited in fully assessing the effects of levosimendan on IL-8 levels.

Data on the effects of levosimendan on IL-17 remain limited. IL-17 is recognized for its dual role—both protective and pathogenic. It contributes to host defense by activating neutrophils and macrophages at sites of infection, thereby promoting anti-microbial responses [30]. However, it is also implicated in the pathogenesis of inflammatory disorders. Studies suggest that IL-17 can enhance immune responses and drive inflammation in early disease stages while potentially suppressing immune activity in later or established disease states [28]. In our study, both low- and high-dose levosimendan significantly elevated IL-17 levels at the 5th hour compared to the sham and sepsis control groups. By the 10th hour, IL-17 levels declined and were comparable to those of the sham group.

There are also conflicting findings regarding the effects of levosimendan in sepsis. Chew et al. [31] investigated its impact on endotoxin-induced pulmonary hypertension and myocardial function in a porcine model. Their results suggested that levosimendan may actually enhance pro-inflammatory responses, notably increasing the expression of IL-6 and IL-8. Furthermore, when administered in conjunction with a standardized vasopressor and fluid resuscitation protocol, levosimendan failed to improve cardiac, renal, or hepatic function in this model of acute porcine endotoxemia. These results contrast with those reported by Boost et al. [32], who found that prophylactic administration of levosimendan in a rat model of non-septic ventilator-induced lung injury (VILI) effectively reduced the pro-inflammatory cytokine response. The measured pro-inflammatory cytokines IL-1 and MIP-2 were significantly reduced in the inhalation group and the intravenous injection group, indicating the anti-inflammatory effects of levosimendan. Our findings showed an early-phase increase in IL-6 levels with both doses. However, in the late phase, IL-6 levels significantly declined, with the reduction being more pronounced in the high-dose group.

Several limitations of this study should be acknowledged. This study only assessed outcomes up to 10 h post-sepsis induction, limiting long-term evaluation. No histopathology was performed to assess organ-specific effects. Additionally, LPS-induced sepsis models do not fully replicate human sepsis complexity. Repeated or prolonged dosing effects of levosimendan were not explored.

## 5. Conclusions

Building on previous findings, our results further support the dose and time-dependent anti-inflammatory effects of levosimendan, particularly its ability to reduce specific pro-inflammatory cytokines, including TNF-α, IL-1β, and MCP-1. We observed a reduction in early acting cytokines following levosimendan treatment, especially at higher doses, supporting the hypothesis of a dose-dependent anti-inflammatory effect. Moreover, the levosimendan-treated groups exhibited a clinically significant reduction in MSS scores at the 10th hour, with the high-dose group showing the most substantial improvement. Our results also reveal changes in IL-6, IL-17, and IL-8 levels, which may exhibit a biphasic response not evident in earlier models. The inclusion of IL-17 and IL-8 findings, alongside established markers such as TNF-α, IL-1β, IL-6, and MCP-1, enhances our understanding of levosimendan’s immunopharmacological profile. These findings emphasize the drug’s therapeutic potential in managing sepsis. However, despite promising experimental outcomes, translating cytokine-targeting therapies from murine models to clinical settings remains a significant challenge due to the complexity of immune interactions in human sepsis.

## Figures and Tables

**Table 1:** Mean cytokine levels measured in the serum of rats in each group.

[ng/L]	Group A	Group B 5th Hour	Group B 10th Hour	Group C 5th Hour	Group C 10th Hour	Group D 5th Hour	Group D 10th Hour
TNF-α	129.46 ± 10.14	212.53 ± 54.28 *	210.27 ± 78.44 *	196.58 ± 19.0	114.78 ± 63.75 ^^Ψ^	223.20 ± 43.28 *	125.42 ± 20.98 ^Ψ^
IL-1β	11.56 ± 0.32	13.06 ± 0.97	13.82 ± 1.19 *	14.83 ± 1.79 *^	13.18 ± 1.58 *^Ψ^	13.61 ± 1.65 *	11.84 ± 0.52 ^^Ψ^
IL-6	3.71 ± 0.21	4.29 ± 1.24	4.60 ±0.22 *	5.13± 1.11 *	4.32 ±1.21	5.87 ± 1.13 ^*	3.78 ± 0.81 ^Ψ^
IL-8	200.37 ± 7.22	205.99 ± 20.95	218.40 ± 32.37	218.90 ± 25.15	229.50 ± 24.34	229.27 ± 36.31	195.32 ± 33.10 ^Ψ^
IL-17	97.99 ± 9.83	117.55 ± 27.47	121.33 ± 25.59	159.93 ± 28.64 *^	103.43 ± 21.39 ^Ψ^	168.19 ± 30.22 *^	106.30 ± 14.86 ^Ψ^
MCP-1	116.34 ± 6.17	186.26 ± 45.25 *	143.79 ± 14.21 ^Ψ^	154.63 ± 37.8 *	171.82 ± 20.51 *	198.77 ± 72.66 *	107.16 ± 18.57 ^Ψ^

Values expressed as mean ± SD. Group A: sham, Group B: sepsis control, Group C: levosimendan 1 mg/kg, Group D: levosimendan 2 mg/kg. *p* < 0.05: * compared to Group A, ^ compared to Group B at the same time point, ^Ψ^ compared to the same group at different time point.

## Data Availability

All data generated as part of this study are included in the article.

## Abbreviations

The following abbreviations are used in this manuscript:

TNF-α	Tumor Necrosis Factor-alpha
IL-1β	Interleukin-1-beta
IL-6	Interleukin-6
L-8	Interleukin-8
IL-17	Interleukin-17
MCP-1	Monocyte Chemoattractant Protein-1
